# Ferro-Phosphas Calcis in Pulmonary Consumption, Etc.

**Published:** 1853-01

**Authors:** H. A. Ramsay

**Affiliations:** Thompson, Columbia Co., Geo.


From the Boston Medical and Surgical Journal.

*Ferro-Phosphas Calcis in Pulmonary Consumption, etc., by* H. A. Ramsay, M. D., Thompson, Columbia Co., Geo.

Dear Sir,—As every man in the profession is honorably and morally bound to contribute his mite in alleviating the ills of the human race, permit me to call your attention to the above preparation in the treatment of tubercular disease. It is true my experience has not been great with the article, as it is confined to but few cases; yet, the happy results I derived in these cases induce me to direct attention to the remedy, that others may enter the field and try its effect. I do not pretend to say it will relieve every case, or even a majority; but that it will benefit many cases, particularly in this climate, I can say with some confidence. I merely desire that others may try it, and confirm or repudiate my restricted experience. I will remark, that I believe climate and its collateral influences, have a controlling effect in the therapeutic management of most affections; consequently, I am impressed that the medicine I suggest may answer better in southern tubercular disease, than in that of New England.

My experience is not extensive enough with the remedy, to say in what peculiar form of the disease it will do best. In its incipiency and decline I have used it with marked advantage. I make it thus: ℞. Phosphas calcis, two parts; phosphas ferri, one part. Dose, ten grains thrice daily, in simple syrup; after five or eight days the dose may be increased to fifteen grains, or more as circumstances may justify. The remedy can be made into a syrup very readily, we imagine, though we have not used it that way. To be effectually serviceable it should be used for some time, and the bowels, if much costive, should be kept open with a warm laxative mixture; if otherwise, they should be restrained with some astringent mixture. The remedy, in my estimation, has all the advantages of the *cod-liver* oil, with none of its disadvantages; and I believe—subject, of course, to farther corroboration—it is destined to supercede all other medicines in the general treatment of tubercular consumption, particularly where abscesses have formed, or in its earliest stages. I send you these views, without going into a disquisition on the theory of the treatment, which would be of no utility to any body. As previously stated, I wish to create no undue hopes, but I respectfully invite the profession at large to try the remedy, and if it proves successful in one instance, or is the means of saving a single life from impending death, I shall be amply compensated for giving this article to your readers.

